# Fibronectin type III domain-containing 4 promotes the migration and differentiation of bovine skeletal muscle-derived satellite cells via focal adhesion kinase

**DOI:** 10.1080/19336918.2020.1810508

**Published:** 2020-09-03

**Authors:** Zhao Wang, Zhiqi Wang, Yusheng Pang, Huili Tong, Yunqin Yan, Shuang Li, Shufeng Li

**Affiliations:** Laboratory of Cellular and Developmental Biology, Life Science College, North-east Agricultural University, Harbin, China

**Keywords:** FNDC4, focal adhesion kinase, migration, differentiation

## Abstract

FNDC4 is an anti-inflammatory factor that alters the activation state of macrophages; it is used to treat colitis in mice. However, its role in muscle formation and mechanism of function remains unknown. We found that FNDC4 promotes the bovine MDSCs migration and differentiation. Furthermore, we reported that it interacts with integrin β1 (ITGβ1). FAK, mediated by ITGβ1, regulates cell migration. Our results found FNDC4 to influence the expression of p-FAK, p-paxillin, and vinculin. Then, overexpressed or added FNDC4 protein could not influence migration and differentiation any more when the activated form of FAK was reduced. Therefore, we concluded that FNDC4 promotes the differentiation and migration of bovine MDSCs via the FAK, mediated by the ITGβ1 receptor.

## Introduction

Bovine muscle-derived satellite cells (MDSCs) are differentiation-capable muscle-derived stem cells located between the sarcolemma and basement membrane [1]. These cells remain inactive under normal conditions and can be triggered to differentiate into skeletal muscle cells under certain conditions [2]. The number of skeletal muscle cells affects the quality of the meat [3]. Activated satellite cells have the ability to proliferate and fuse with each other to form myotubes. This process needs the long-distance migration of cells involved in. And the myoutubes further mature to form muscle fibers and participate in the generation of muscles [4]. It is well known that cell migration and differentiation are an inseparable processes [5] and it were affected by many factors, such as some transmembrane protein and extracellular matrix [6].

Fibronectin type III domain-containing 4 (FNDC4) is a member of the fibronectin type III domain family of proteins [7]. FNDC4 is a membrane protein consisting of a C-terminal hydrophobic domain, a type III fibronectin domain, and an N-terminal membrane signaling region. The N-terminus (extracellular region) can be shredded to release the extracellular portion of the protein [8]. The fibronectin type III domain is an evolutionary conserved domain in animal proteins [9]. FNDC4 is a secreted factor sharing high homology with FNDC5, an exercise-induced myokine irisin [8–10]. Irisin is secreted as a cleaved form of FNDC5 [11] and was identified as a myokine secreted by contracting skeletal muscle, possibly mediating some exercise health benefits via ‘browning’ of white adipose tissue [12]. It has been reported that irisin could target osteoblasts and promote osteoblast differentiation [13]. Our previous high-throughput sequencing analysis, we found that FNDC4 expression increases significantly during the differentiation of bovine MDSCs [14]. A recent study has attributed the therapeutic effect of FNDC4 in colitis to its inhibitory effect on macrophages [8]. Lv et al. speculated that FNDC4 could suppress osteoclast formation via the NF-κB pathway [15,16]. However, there is no research on FNDC4 in bovine MDSCs migration and differentiation.

In this study, we show that the FNDC4 expression affects the migration and differentiation of bovine MDSCs. The results of mass spectrometry and co-immunoprecipitation showed that FNDC4, which has an Arg-Gly-Asp (RGD) module within the FNIII domain [8], can interact with ITGβ1. The spliced form of FNDC4 is similar to that of FNDC5; therefore, we hypothesized that cleaved FNDC4 is also a factor secreted by muscle cells and regulates muscle migration and differentiation via ITGβ1-mediated FAK. Our results reveal, for the first time, that the cytokine FNDC4 secreted by the muscle promotes the migration and differentiation of bovine skeletal muscle-derived satellite cells. Moreover, we had studied its mechanism of function. Our research is important for further clarification of the molecular mechanisms of muscle development and the improvement of animal meat quality.

## Materials and methods

### Experimental cells

Skeletal muscle tissues were collected from newborn calves after obtaining approval from the Animal Welfare Committee of Northeast Agricultural University, Heilongjiang Province, China. Skeletal muscle tissues were pooled and finely minced. Subsequently, they were digested by treatment with 0.2% collagenase XI (Sigma-Aldrich, St. Louis, MO, USA) for 2 h, followed by treatment with 0.25% trypsin (Sigma) for 30 min. The isolated bovine MDSCs were cultured in high-glucose Dulbecco’s Modified Eagle’s Medium (DMEM) containing 10% FBS, 100 U/mL penicillin, and 100 μg/ml streptomycin at 37°C and 5% CO_2_ in a humidified atmosphere.

### MDSCs culture and differentiation

MDSCs were maintained in DMEM (Gibco, Grand Island, NY, USA) with 10% FBS (Gibco), 100 U/mlpenicillin (sigma) and 100 μg/ml streptomycin (sigma). When cells reached 60–80% confluence, the medium was switched to differentiation medium, which consisted of 2% horse serum, 100 U/mlpenicillin and 100 μg/ml streptomycin in DMEM for 48 h or 72 h.

### Antibodies and chemicals

The anti-FNDC4, anti-MYOG and anti-desmin were from Bioss Biotechnology. The anti-GAPDH, anti-Phospho-paxillin (Tyr118), anti-paxillin, anti-Phospho-FAK (Tyr925), anti-FAK, anti-Vinculin and anti-ITGβ1 were purchased from Cell Signaling. The anti-6× his and anti-IgG were from Santa Cruze Biotechnology. The secondary antibodies were HRP-labeled and FITC-labeled were from Beijing Biosynthesis Biotechnology Co., Ltd. PF562271 was from Selleck and used concentration for 10 nM/ml to treat cells.

### Constructed and screened plasmid vectors

To examine the effect of FNDC4 on the differentiation of bovine MDSCs, an overexpression vector for FNDC4 was constructed. The primers for FNDC4 were as follows: Sense, TCCCAGCGGACTCGGT; Anti-sense, ATTGATGGATGGTGACTTT. HindIII and EcoRI restriction sites on both sides and a ligase to connect to pcDNA3.1^+^ were added. Next, Baml and EcoRl were used to digest pcDNA3.1^+^ + FNDC4 plasmid, and then the production was connected to pCMV-his-N to obtain pCMV-his-N-FNDC4. MDSCs were plated in six-well plates 24 h before transfection with polyethylenimine (PEI) (Sigma-Aldrich). Cells were transfected with the pcDNA3.1^+^ + FNDC4 plasmid (pcDNA3.1^+^, Addgene #V790-20, Middlesex, UK; 2 µg).

Bovine MDSCs were cultured for 24 h and then transfected with 50 nM siRNA for FNDC4 and negative control by using Lipofectamine 2000 (Invitrogen, Carlsbad, CA, USA), according to the manufacturer’s recommended protocol. The sense sequences of siRNAs used were: Negative control siRNA (siNegative), 5ʹ-UUCUCCGAACGUGUCACGUTT-3ʹ; siFNDC4-198: 5ʹ-UCACUCACCUCAGAGCCAATT-3ʹ; siFNDC4-483: 5ʹ-GCCCAGGUGACAUCACAGUTT-3ʹ; siFNDC4-589: 5ʹ-GCUAUUCUGCCGUCAGUAUTT-3ʹ. Protein expression was measured 72 h after transfection by Western blotting.

### Mass spectrometry and co-immunoprecipitation

MDSCs were plated in a 10-cm dish 24 h before transfection with PEI (Sigma-Aldrich). Cells were transfected with the pcDNA3.1^+^ + FNDC4 plasmid and cultured with differentiation medium (2% horse serum) for 5 days. After differentiation, cells were lysed for 3 h at 4°C in RIPA buffer (150 mM NaCl, 50 mM Tris, 1 mM EDTA). The lysate was split into two equal parts. At least 400 µL of lysate was mixed with 2 µg of anti-FNDC4 or anti-ITGβ1 and anti-IgG. Immunoprecipitation was performed overnight on a rotator at 4°C, followed by 4-times washing with 40 µL of BSA-blocked protein A-agarose beads (Sigma) and RIPA buffer at 12000 xg for 5 min. The protein lysates incubated with the antibody overnight were combined with protein A-agarose beads and incubated for 4 h on a rotator at 4°C. The final pellet was resuspended in Laemmli loading buffer and boiled. Samples were analyzed by SDS-page, the whole gel was stained with Coomassie blue, and then compared with the IgG group. The specific band was cut and sent to Zhongke New Life Biotechnology Co., Ltd. (Shanghai, China) for mass spectrometry. Moreover, samples were analyzed by western blotting to detect protein interaction.

### Isolation and purification of FNDC4 extracellular protein

MDSCs were transfected with the pCMV-his-N-FNDC4 plasmid. Thereafter, the culture solution was collected and the secreted FNDC4 protein was isolated and purified using a His·Bind Purification Kit (Millipore). Next, the protein was concentrated using a 15-mL ultrafiltration tube (molecular weight cutoff 10 kDa) (Millipore) and then the concentration was measured using a BCA Protein Assay Kit (CWBIO, Beijing, China). The protein purity was measured using a Coomassie blue stained gel after SDS-PAGE with an FNDC4 antibody and histidine-tagged antibody.

### Western blot assay

Cellular proteins were extracted from bovine MDSCs, separated by 15% SDS-polyacrylamide gel electrophoresis (SDS-PAGE), and transferred to a PVDF membrane (Millipore). The PVDF membrane was incubated in blocking solution (PBST containing 5% skim milk powder) at 37°C for 1 h, followed by incubation with specific primary antibodies at 37°C for 1 h. The proteins were visualized using the Super ECL Plus Detection Kit (Applygen Technologies Inc., Beijing, China) using the Minichemi Chemiluminescence Imaging Instrument (Beijing Sage Creation Science Co., Beijing, China) according to the manufacturer’s instructions. A densitometry analysis was performed using SageCapture™ software (Beijing Sage Creation Science Co.).

### Immunofluorescence

Cells on coverslips were washed twice with PBS and fixed in cold methanol for 20 min. Then, the cells were incubated for 1 h with PBST (0.5% Triton X-100 in PBS) containing 5% bovine serum albumin (BSA) and incubated with a primary antibody specific to desmin or FNDC4 at an appropriate dilution for 60 min at 37°C. The cells were then rinsed thrice with PBST and incubated with the corresponding FITC-conjugated secondary antibody for 60 min at 37°C. The cells were rinsed thrice with PBST and incubated DAPI for 5 min to visualize the nuclei. The cells were again rinsed thrice with PBST before observation. The images were obtained from random 5 fields of view in each treatment group and counted. The myotubes fusion rate was assessed by counting the number of nuclei in differentiated myotubes (>2myonuclei) as a percentage of the total number (mononucleated).

### Cell scratch assays

MDSCs were seeded on 12-well plates (Costar Corning, Inc., Corning, NY, USA) at a cell density of 5 × 10^5^ cells/well in complete media; pcDNA3.1^+^+FNDC4 transfection was performed using PEI, and siFNDC4-589 transfection was performed using Lipofectamine 2000. At 0 h, a 200-µL pipette tip was used to scratch the confluent monolayer. The medium was replaced with differentiation medium (2% horse serum). At 48 h, images of scratches were acquired using an Olympus 1 × 71 microscope and Olympus DP72 camera (Olympus America, Inc., Center Valley, PA, USA). Using ImageJ (National Institutes of Health, Bethesda, MD, USA), the area between cells was measured to analyze migration efficiency. Eight measurements were completed for each scratch at each time point. Four scratches were used for each experiment. The experiments were repeated with three separate MDSC preparations.

### Statistical analyses

Grayscale scanning of protein bands in western blots was performed using the ImageJ software (National Institutes of Health), All results are expressed as means ± SEM. Representative bands were selected from independent western blotting experiments. Statistical significance was determined by Students' t-test and Bonferroni’s post-hoc tests using GraphPad Prism (GraphPad Software, La Jolla, CA, USA). Statistical analyses were performed using SPSS software (IBM Corp., Armonk, NY, USA). Differences were regarded as significant at a P-value <0.05.

## Results

### FNDC4 expression during bovine MDSC differentiation

**Figure 1.**
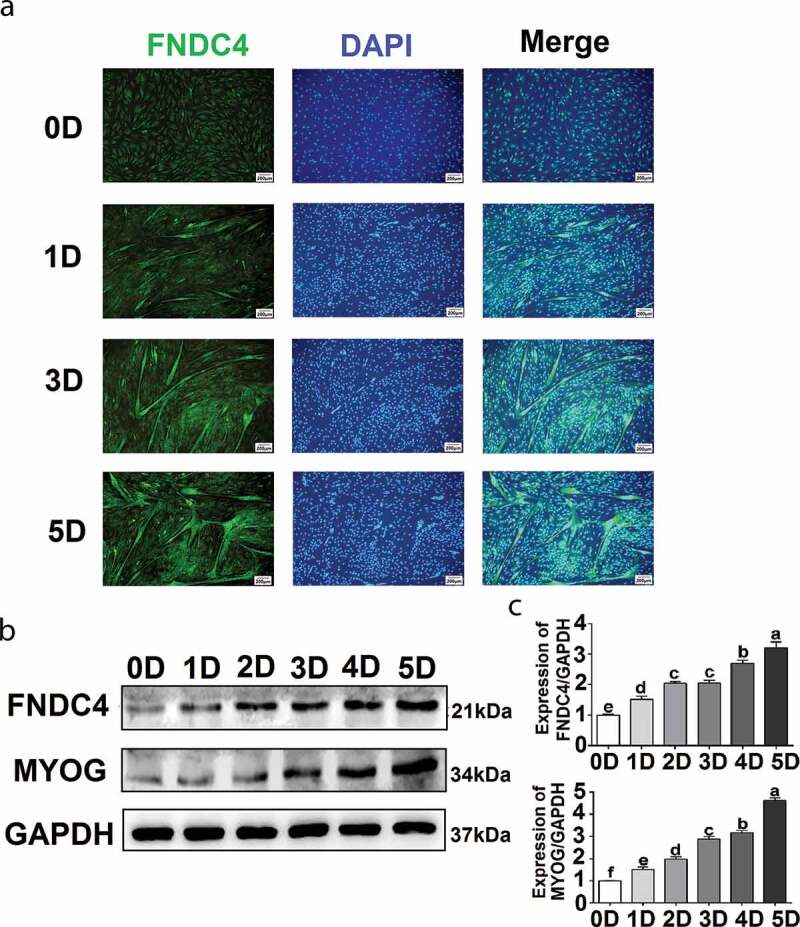
Fibronectin type III domain-containing 4 (FNDC4) expression during bovine skeletal muscle-derived satellite cell (MDSC) differentiation. (a) MDSCs were induced to differentiate for various lengths of time, and immunofluorescence analyses were performed to determine FNDC4 expression and to visualize total DNA (DAPI). (b) FNDC4 and MYOG expression in MDSCs at days 0 (0D), 1 (1D), 2 (2D), 3 (3D), 4 (4D), and 5 (5D) after the initiation of differentiation. (c–d) Quantification of FNDC4 and MYOG expression. ab, *P* < 0.01, compared with the D0 group (*n* = 3), magnification, 200×.

Immunofluorescence staining of FNDC4, which appeared as a diffuse signal in the cytoplasm, did not reveal any myotubes when bovine MDSCs were cultivated in growth medium. After cultivation in differentiation medium for 1, 2, 3, 4, or 5 days (D1, D2, D3, D4, or D5, respectively), short myotubes formed gradually, multinucleated myotubes were observed, and the fluorescent intensity of FNDC4 increased and was localized to the cytoplasm of the multinucleated myotubes at D1, D3, and D5 (Figure 1a). Protein levels were analyzed at D1, D2, D3, D4, and D5 in MDSCs. The FNDC4 level increased gradually with differentiation time (Figure 1b). The FNDC4 level increased 1.17-fold at D1 (*P* < 0.05), 1.57-fold at D2 (*P* < 0.01), 1.61-fold at D3 (*P* < 0.01), 1.78-fold at D4 (*P* < 0.01), and 2.05-fold at D5 (*P* < 0.01) compared to the levels observed in undifferentiated bovine MDSCs (D0) (Figure 1c). In addition, MYOG levels increased gradually during the successive differentiation stages in the same samples (Figure 1d).

### Effect of FNDC4 on the migration and differentiation of bovine MDSCs

To study the effect of FNDC4 expression on the differentiation of bovine MDSCs, we overexpressed or inhibited FNDC4 expression. The cells were allowed to differentiate for 72 h after transfection, and the expression levels of FNDC4 and MYOG were detected by western blot analysis.

**Figure 2.**
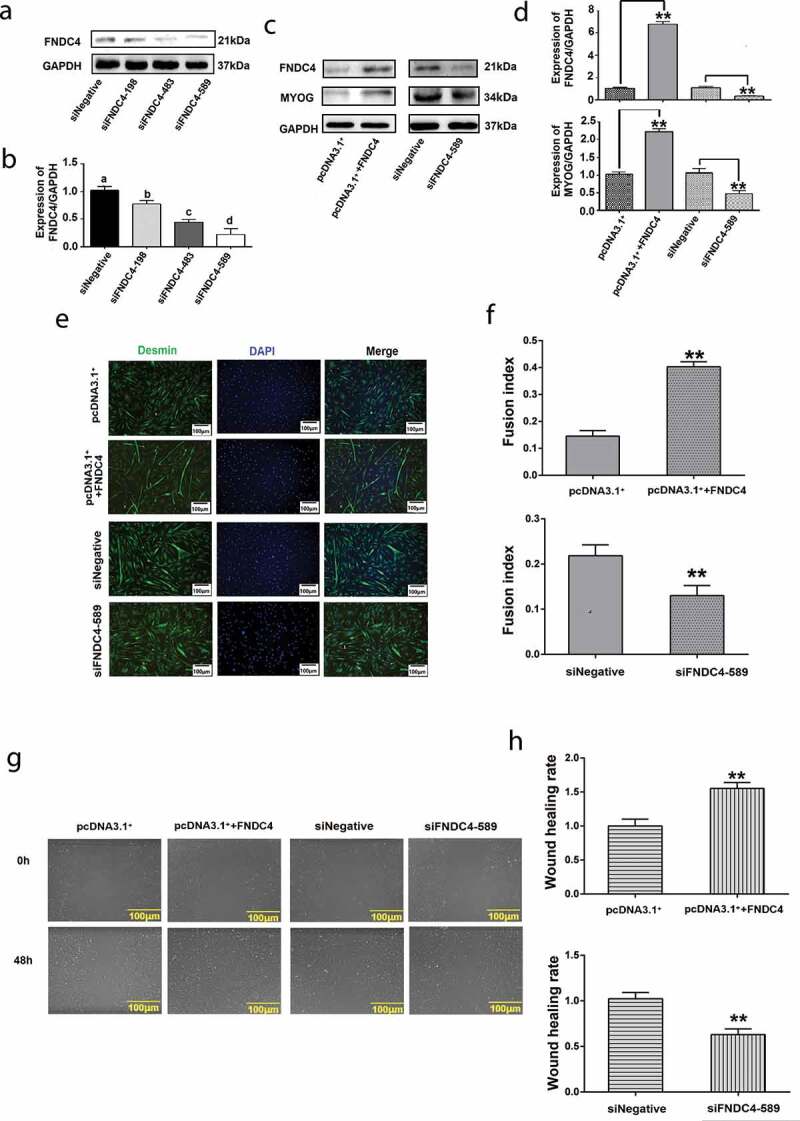
Effect of fibronectin type III domain-containing 4 (FNDC4) on the migration and differentiation of bovine muscle-derived satellite cells (MDSCs). (a) FNDC4 expression in MDSCs after transfection with three siRNAs and siRNA with a random sequence (siNegative). (b) FNDC4 expression analysis. (c) FNDC4 and MYOG levels in MDSCs after transfection with pcDNA3.1^+^ + FNDC4; pcDNA3.1^+^ was used as the control group or cells were transfected with siFNDC4-589, and siNegative was used as the control group for 72 h. (d) Statistical analysis of the results presented in (C). (e) Immunofluorescence detection of desmin expression in MDSCs transfected with pcDNA3.1^+^ + FNDC4; pcDNA3.1^+^ was used in the control group. Cells were transfected with siFNDC4-589 and siNegative was used as control group. (f) Quantitative analysis of myotube fusion index of desmin-positive cells presented in (E). (g) Cell scratch assay. MDSCs after transfection with pcDNA3.1^+^ + FNDC4; pcDNA3.1^+^ was used as control group. MDSCs were transfected with siFNDC4-589, and an irrelevant siRNA (siNegative) was used as control group. 0 h represents cells before migration, 48 h represents cells that was differentiated after transfected vectors. (h) Quantification of cell migration analysis after overexpress or inhibition of FNDC4. **P < 0.01, compared to cells in control group (*n* = 3), magnification, 100×.

Firstly, we screened vectors that inhibited the expression of FNDC4 by western blotting. The result showed that the expression levels of the FNDC4 protein in bovine MDSCs transfected with siFNDC4-198, siFNDC483, and siFNDC4-589 plasmid vectors differed by 0.82-fold (*P* < 0.05), 0.52-fold (*P* < 0.01), and 0.31-fold (*P* < 0.01) from those of the negative control group (Figure 2a and b). Therefore, siFNDC4-589 was selected for subsequent experiments.

After transfection with pcDNA3.1^+^ + FNDC4, the FNDC4 levels were 6.83-fold (*P* < 0.01) higher than those in the control group. MYOG levels were 2.23-fold (*P* < 0.01) higher at 72 h following the increase in FNDC4 levels. On the contrary, MYOG levels decreased by 51% (*P* < 0.01) at 72 h, following the decrease in FNDC4 levels (Figure 2c and d). Alterations in the myotube fusion rate during *in vitro* differentiation were observed by desmin immunofluorescence staining. Myotube formation was induced; the myotube-fusion index increased by 25% (*P* < 0.01) after transfection with pcDNA3.1^+^ + FNDC4. After inhibition of FNDC4, the myotube formation was suppressed and the myotube-fusion index decreased by 47% (*P* < 0.05) (Figure 2e and f).

A cell scratch assay showed that the remaining area between cells was reduced after transfection with the FNDC4 overexpression vector and the wound healing rate increased significantly. The opposite phenomenon occurs after inhibiting the expression of FNDC4 (Figure 2g and h). These results indicating that FNDC4 promotes the migration and differentiation of bovine MDSCs.

### FNDC4 combines with ITGβ1 and affect FAK

We found that the overexpression and inhibition of FNDC4 affected cell differentiation and migration, but the mechanism is unclear. We used mass spectrometry to analyze proteins that interact with FNDC4. The results of mass spectrometry are shown in supplementary materials 1. We identified ITGβ1 and speculated that FNDC4 may act as a ligand for ITGβ1. To confirm this, we used co-immunoprecipitation (Co-IP) assay.

**Figure 3.**
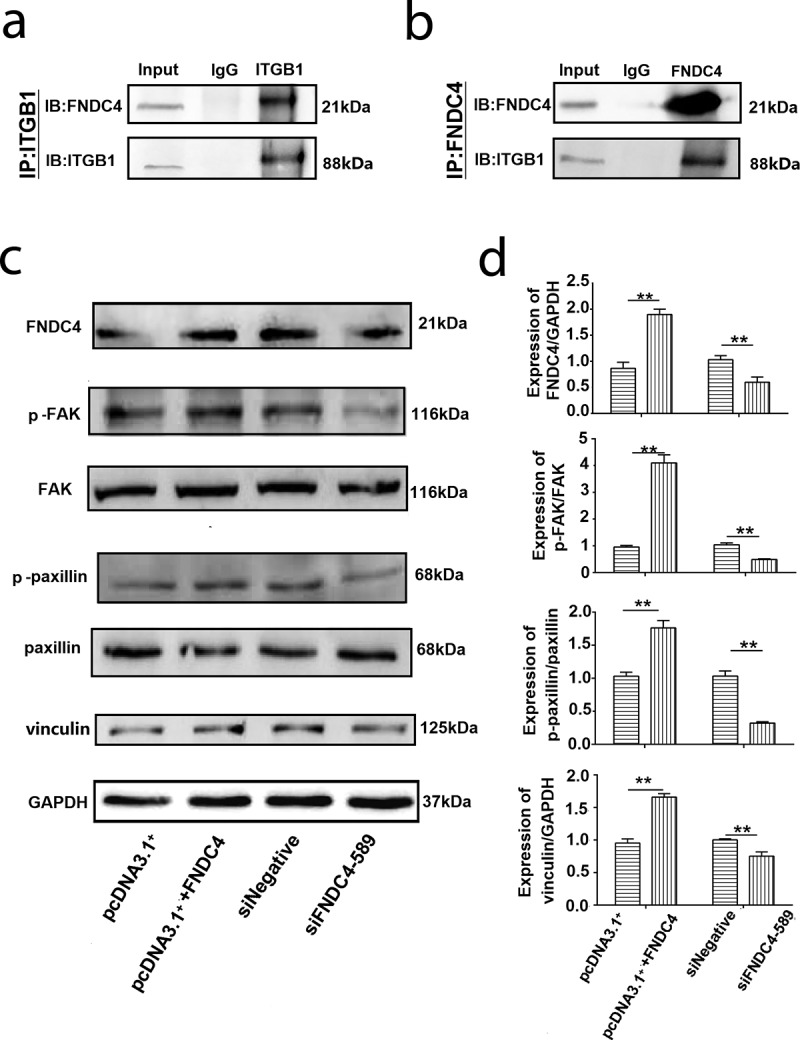
FNDC4 interact with ITGβ1 and affect FAK. (a) Precipitation of FNDC4 using an ITGβ1 antibody, with ITGβ1 as a control group. (b) Precipitation of FNDC4 using an ITGβ1 antibody, with FNDC4 as a control group. (c) FNDC4, p-FAK, p-paxillin and vinculin levels in MDSCs after transfection with pcDNA3.1^+^ + FNDC4; pcDNA3.1^+^ was used as the control group or MDSCs were transfected with siFNDC4-589, and siNegative was used as the control group for 72 h. (d) Statistical analysis of the results presented in (C). **P < 0.01, compared to cells in control group (*n* = 3), magnification, 100×.

Bovine MDSCs were transfected with an FNDC4 overexpression vector and differentiated in culture. After lysing cells with RIPA lysate, they were bound overnight to FNDC4 and ITGβ1 antibodies. Western blotting showed that FNDC4 can specifically bind to ITGB1 using FNDC4 as a control group and that ITGβ1 can bind to FNDC4 using ITGβ1 as a control group (Figure 3a and b). These results indicated that FNDC4 can bind to ITGβ1, and thus, we speculated that FNDC4 plays a role in the differentiation of bovine MDSCs via FAK that was mediated by ITGβ1.

To evaluate the effect of FNDC4 expression on the FAK activity during bovine MDSCs differentiation, we detected the expression of p-FAK, p-paxillin and vinculin after overexpression or inhibition of FNDC4. The results showed that the levels of p-FAK, p-paxillin and vinculin in cells which were overexpressed FNDC4 were higher than those in the control group significantly. However, p-FAK, p-paxillin and vinculin were decreased after interference with the expression of FNDC4.

### Effect of FAK inhibitor PF562271 on FNDC4 promoting migration and differentiation of bovine MDSCs

**Figure 4.**
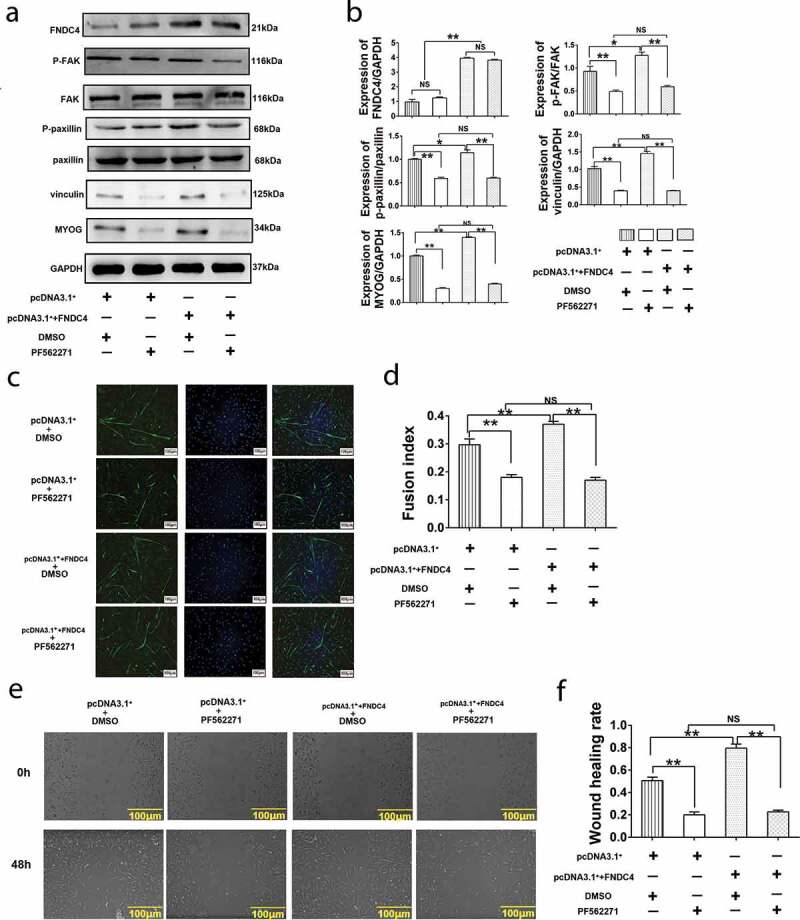
Effects of inhibit FAK on fibronectin type III domain-containing 4 (FNDC4) promote migration and differentiation of bovine MDSCs. (a) MDSCs were transfected with pcDNA3.1^+^ and treated with DMSO; MDSCs were transfected with pcDNA3.1^+^ and treated with FAK inhibitor (PF562271); MDSCs were transfected with pcDNA3.1^+^ + FNDC4 and treated with DMSO; MDSCs were transfected with pcDNA3.1^+^ + FNDC4 and added with PF562271. Under these conditions, FNDC4, p-FAK, p-paxillin, vinculin and MYOG levels were detected. (b) Statistical analysis of the results presented in (A). (c) The cells under the same conditions with (A). Immunofluorescence detection of desmin expression in muscle-derived satellite cells (MDSCs) were detected. Magnification, 100×. (d) Quantitative analysis of the myotube fusion index of desmin-positive cells presented in (C). (e) Cell scratch assay. MDSCs under the same conditions with (A). 0 h represents cells before migration, 48 h represents cells that was differentiated after transfected vectors. (f) Quantification of cell migration analysis in (E). Values represent the mean percentage of nuclei in desmin-positive cells ± SEM. *P < 0.05, **P < 0.01, NS: No significant difference compared to cells in control group (*n* = 3), magnification, 100×.

In order to detect whether FNDC4 promotes the migration and differentiation of bovine muscle satellite cells through FAK, we used PEI to transfect cells with an overexpression plasmid vector and control plasmid. Then, the FAK inhibitor PF562271 was added into the differentiation culture medium to differentiate the cultured bovine MDSCs for 72 h, and then the related proteins and MYOG were detected. The results showed that the expression level of p-FAK, p-paxillin vinculin and MYOGdecreased (*P* < 0.01) after added PF562271. After transfection of the overexpression vector, the levels of the above protein levels were elevated (*P* < 0.01). Addition of PF562271 following transfection of the overexpression vector decreased the expression levels of these proteins compared with control group and no significant difference with added PF562271 alone (*P* < 0.01) (Figure 4a and b). Immunofluorescence results showed that after the addition of PF562271, the myotube fusion rate decreased (*P* < 0.01). However, after overexpression of FNDC4, the myotube fusion rate increased. Addition of PF562271 following FNDC4 overexpression decreased the myotube fusion rate (*P* < 0.01) (Figure 4c and d). Cell scratch assay showed that the wound healing rate decreased significantly after added PF562271 compared with control group. Overexpression FNDC4 caused increased wound healing rate. However, inhibition of FAK after overexpression of FNDC4 does not increase wound healing rate (Figure 4e and f). These result addition of FAK inhibitors block the promotion of FNDC4 on migration and differentiation of bovine MDSCs.

### Effect of extracellular cleaved FNDC4 protein on the migration and differentiation of bovine MDSCs

**Figure 5.**
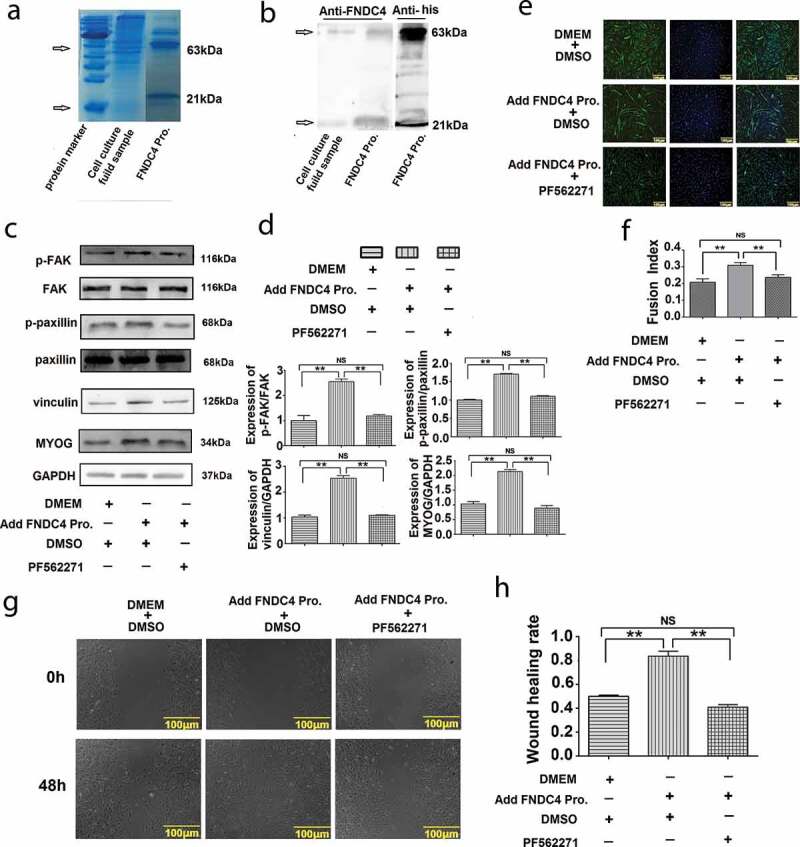
Summary of isolation and purification of extracellular protein of fibronectin type III domain-containing 4 (FNDC4) and the effect of FNDC4 addition on muscle-derived satellite cells (MDSC) migration and differentiation *in vitro*. (a) Coomassie brilliant blue staining; cell culture medium was used as a control, and purified FNDC4 protein was isolated as an experimental sample. (b) Detection of isolated and purified FNDC4 protein by FNDC4 antibody and histidine-tagged antibody; cell culture medium was used as a control. (c) The effect of FNDC4 extracellular domain on FAK, paxillin, vinculin and MYOG in *vitro*. After adding FNDC4 protein, the signal pathway inhibitor PF562271 was added to the experimental group, and DMSO was added to the control group. (d) Statistical analysis of the results presented in (C). (e) Immunofluorescence was used to detect the effect of FNDC4 extracellular domain on desmin. After adding FNDC4 protein (DMEM as control), the signal pathway inhibitor PF562271 was added to the experimental group, and DMSO was added to the control group. (f) Quantitative analysis of the myotube fusion index of desmin-positive cells presented in(E). (g) The cells were under the same condition with (E). The rate of cell migration was detected. (h) Quantification of cell migration analysis in (E). Values represent the mean percentage of nuclei in desmin-positive cells ± SEM. *P < 0.05, **P < 0.01, NS: No significant difference compared to cells in control group (*n* = 3), magnification, 100×.

The FNDC4 protein secretion vector pCMV-his-N-FNDC4 was transfected into bovine MDSCs for 5 days. The culture medium was recovered through a Ni-resin chromatography column and concentrated by an ultrafiltration tube. The purity of the recovered protein was detected by SDS-PAGE gel electrophoresis. As shown in Figure 5a, we found a band of interest near the target protein (21 kDa) and a band around 63 kDa. This was detected by FNDC4 antibody and a histidine-tagged antibody. The proteins were detected by both antibodies (Figure 5b). After the addition of FNDC4 extracellular secretory protein *in vitro*, the expression of phosphorylation level of FAK and paxillin, vinculin and MYOG were increased (*P* < 0.01). Addition of PF562271 following the addition of FNDC4 protein inhibited the expression of the above proteins (*P* < 0.01) (Figure 5c and d). The immunofluorescence results showed that the myotube fusion rate increased after the addition of FNDC4 protein (*P* < 0.01); however, the myotube fusion rate decreased when PF562271 was added following the addition of FNDC4 protein (*P* < 0.01) (Figure 5e and f).

Cell scratch assay showed that the wound healing rate increased after added FNDC4 protein (Figure 5g and h).

## Discussion

Skeletal muscle content is an important property in animal husbandry. Mononuclear cells will proliferation and migrate to form multinucleated cells, which lead to cell differentiation, forming multiple hematocles, and then forming muscle tissue [17]. Often, these muscle cells migrate long distances prior to fusion [18]. These processes were regulated by many factors such as MYOG [19]. Desmin, the muscle-specific intermediate filament protein, is one of the earliest appearing myogenic markers in both skeletal and heart muscles [20]. Therefore, it is important to find genes that affect muscle migration and differentiation. FNDC4 is a member of the fibronectin type III domain family of proteins and shows high homology with irisin [8]. Colaianni et al. showed that irisin secreted from myoblasts could directly target osteoblasts and thus enhance their differentiation [21]. The research further proved that irisin could promote osteoblast proliferation and differentiation via the p38/MAPK signaling pathways [13]. In our experiment, suggesting that FNDC4 involved in differentiation of bovine MDSCs affects the migration and differentiation of bovine MDSCs.

Furthermore, we demonstrated that FNDC4 can interact with ITGβ1. Integrins are transmembrane heteromeric receptors that mediate interactions between cells and the extracellular matrix [22]. ITGβ1 is the most abundantly expressed β subunit and is found in almost all cell types in the body [23]. It acts as a membrane molecule that mediates signal transduction via a unique pathway; a large number of cell phenotypes are regulated by integrins, including cell growth, differentiation, migration, invasion, and apoptosis [24]. The early stages of cell migration and invasion involve ruffling of the plasma membrane into distinct structures, such as filopodia or lamellipodia, which contain bundles of actin filaments and associated proteins [25]. The activation of β 1 increases cell traction forces [26]. There were reported that β1 integrins in myoblasts regulate skeletal muscle fiber development [27]. So we detected the FAK which was mediated ITGβ1.

Myosin-mediated contractility regulates adhesion through the recruitment of signaling proteins to focal adhesions, which are central checkpoints in transforming extracellular mechanical cues into cellular responses and in transmitting contractile forces to the extracellular matrix [28]. FAK is an intracellular tyrosine kinase with roles in cell motility, cell migration, and cell survival [29]. Paxillin mediates cell movement by recruiting cytoskeletal elements and signaling molecules involved in cell attachment, spreading, and migration [30]. The FAK/paxillin interaction is modulated by the cytoskeletal adapter protein vinculin via the extracellular signal-regulated kinase (ERK) pathway [31]. Vinculin-mediated modulation of paxillin-FAK interactions regulates ERK to control survival and motility [32]. Signaling downstream of these FA proteins impacts proliferation, differentiation, migration, and other cellular functions [33–36]. Therefore, FNDC4 affects the FAK to influence cell migration, thereby influencing the differentiation of bovine MDSCs.

**Figure 6.**
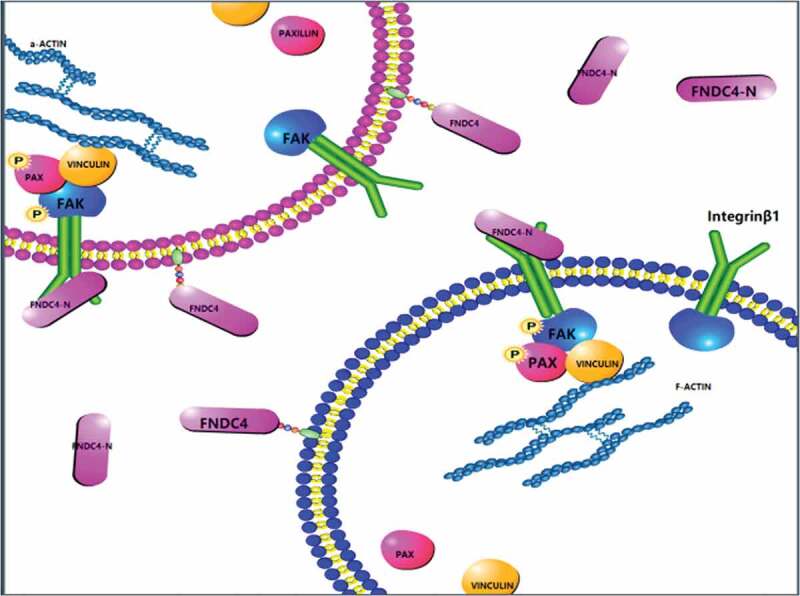
Schematic diagram of the molecular mechanism of FNDC4 promoting the migration and differentiation of bovine MDSCs. During bovine MDSCs differentiation, the extracellular domain of FNDC4 is cleaved and interacts with ITGβ1 which was on the cell membrane, affecting the activity of FAK and the formation of complexes, which in turn affects cell migration.

Our results provide new ideas for the study of the relationship between extracellular secretory proteins and membrane receptors. Here, we showed that FNDC4 affected the expression levels of p-FAK, p-paxillin, and vinculin. Next, to explore whether FNDC4 works through FAK, FAK inhibitor (PF-562271) was used. PF-562271 is a potent, ATP-competitive, reversible inhibitor of FAK and its Pyk2 catalytic activity [37]. Bovine MDSCs were also treated with the FAK signaling pathway inhibitor PF562271; meanwhile, overexpression of FNDC4 or in vitro addition of FNDC4 secretion protein did not increase the expression of p-FAK, p-paxillin, vinculin and MYOG. This not only demonstrates that FNDC4 promotes differentiation and migration of bovine muscle satellite cells via FAK, and demonstrates that the extracellular domain of FNDC4 is functional. Taken together, we demonstrated that FNDC4 binds to the integrin β1 and regulates the differentiation and migration of bovine MDSCs via the FAK (Figure 6).

Additionally, these results provide insight into the relationship between cell migration and differentiation. The results of this study enrich our knowledge of skeletal muscle cell differentiation and regeneration, provide an important basis for animal nutrition and livestock meat quality improvement, and may also provide new targets for transgenic beef cattle.

## Supplementary Material

Supplemental Material

## References

[1] Jang Y, Sinha M, Cerletti M, et al. Skeletal muscle stem cells: effects of aging and metabolism on muscle regenerative function. Cold Spring Harb Symp Quant Biol. 2011;76:101–111. 21960527 10.1101/sqb.2011.76.010652

[2] Dumont NA, Bentizinger CF, Sincennes MC, et al. Satellite cells and skeletal muscle regeneration. Compr Physiol. 2015;5:1027–1059. 26140708 10.1002/cphy.c140068

[3] Charge SB, Rudnicki MA. Cellular and molecular regulation of muscle regeneration. Physiol Rev. 2004;84(1):209–238. 14715915 10.1152/physrev.00019.2003

[4] Parker MH, Seale P, Rudnicki MA. Looking back to the embryo: defining transcriptional networks in adult myogenesis. Nat Rev Genet. 2003;4(7):497–507. 12838342 10.1038/nrg1109

[5] Lin F, Zhang W, Xue D, et al. Signaling pathways involved in the effects of HMGB1 on mesenchymal stem cell migration and osteoblastic differentiation. Int J Mol Med. 2016;37(3):789–797. 26846297 10.3892/ijmm.2016.2479

[6] Pang Y, Zhang Z, Wang Z, et al. Platelet endothelial aggregation receptor-1 regulates bovine muscle satellite cell migration and differentiation via integrin beta-1 and focal adhesion kinase. Cell Adh Migr. 2019;13(1):192–202. 31096840 10.1080/19336918.2019.1619434 PMC6550786

[7] Teufel A, Malik N, Mukhopadhyay M, et al. Frcp1 and Frcp2, two novel fibronectin type III repeat containing genes. Gene. 2002;297(1–2):79–83. 12384288 10.1016/s0378-1119(02)00828-4

[8] Bosma M, Gerling M, Pasto J, et al. FNDC4 acts as an anti-inflammatory factor on macrophages and improves colitis in mice. Nat Commun. 2016;7:11314. 27066907 10.1038/ncomms11314 PMC4832079

[9] Bazan JF. Structural design and molecular evolution of a cytokine receptor superfamily. Proc Natl Acad Sci U S A. 1990;87(18):6934–6938. 2169613 10.1073/pnas.87.18.6934 PMC54656

[10] Boström P, Wu J, Jedrychowski MP, et al. A PGC1-α-dependent myokine that drives brown-fat-like development of white fat and thermogenesis. Nature. 2012;481(7382):463–468. 22237023 10.1038/nature10777 PMC3522098

[11] Du XL, Jiang WX, Lv ZT. Lower circulating irisin level in patients with diabetes mellitus: a systematic review and meta-analysis. Horm Metab Res. 2016;48(10):644–652. 27300472 10.1055/s-0042-108730

[12] Kurdiova T, Balaz M, Vician M, et al. Effects of obesity, diabetes and exercise on Fndc5 gene expression and irisin release in human skeletal muscle and adipose tissue: in vivo and in vitro studies. J Physiol. 2014;592(5):1091–1107. 24297848 10.1113/jphysiol.2013.264655 PMC3948565

[13] Qiao XY, Nie Y, Ma YX, et al. Irisin promotes osteoblast proliferation and differentiation via activating the MAP kinase signaling pathways. Sci Rep. 2016;6:18732. 26738434 10.1038/srep18732 PMC4704023

[14] Tong HL, Yin HY, Zhang WW, et al. Transcriptional profiling of bovine muscle-derived satellite cells during differentiation in vitro by high throughput RNA sequencing. Cell Mol Biol Lett. 2015;20(3):351–373. 26208385 10.1515/cmble-2015-0019

[15] Lv ZT, Liang S, Chen K, et al. FNDC4 inhibits RANKL-induced osteoclast formation by suppressing NF- B activation and CXCL10 expression. Biomed Res Int. 2018;2018:3936257. 29977911 10.1155/2018/3936257 PMC5998196

[16] Chicurel ME, Singer RH, Meyer CJ, et al. Integrin binding and mechanical tension induce movement of mRNA and ribosomes to focal adhesions. Nature. 1998;392:730–733. 9565036 10.1038/33719

[17] Sun H, Cao Y, Zhao Y, et al. MicroRNAs in skeletal muscle differentiation. Semin Cell Dev Biol. 2017;72:67–76. 29102719 10.1016/j.semcdb.2017.10.032

[18] Abmayr SM, Pavlath GK. Myoblast fusion: lessons from flies and mice. Development. 2012;139(4):641–656. 22274696 10.1242/dev.068353 PMC3265056

[19] Tesař V, Zima T. Recent progress in the pathogenesis of nephrotic proteinuria. Crit Rev Clin Lab Sci. 2008;45(2):139–220. 18415815 10.1080/10408360801934865

[20] Paulin D, Desmin: LZ. A major intermediate filament protein essential for the structural integrity and function of muscle. Exp Cell Res. 2004;301(1):1–7. 15501438 10.1016/j.yexcr.2004.08.004

[21] Colaianni G, Cuscito C, Mongelli T, et al. Irisin enhances osteoblast differentiation in vitro. Int J Endocrinol. 2014;2014:902186. 24723951 10.1155/2014/902186 PMC3960733

[22] McHugh KP, Hodivala-Dilke K, Zheng MH, et al. Mice lacking β3 integrins are osteosclerotic because of dysfunctional osteoclasts. J Clin Invest. 2000;105(4):433–440. 10683372 10.1172/JCI8905 PMC289172

[23] Kreidberg JA, Symons JM. Integrins in kidney development, function, and disease. Am J Physiol Renal Physiol. 2000;279(2):F233–42. 10919841 10.1152/ajprenal.2000.279.2.F233

[24] Schwander M, Shirasaki R, Pfaff SL, et al. Beta1 integrins in muscle, but not in motor neurons, are required for skeletal muscle innervation. J Neurosci. 2004;24(37):8181–8191. 15371519 10.1523/JNEUROSCI.1345-04.2004 PMC6729792

[25] Chiu KY, Wu CC, Chia CH, et al. Inhibition of growth, migration and invasion of human bladder cancer cells by antrocin, a sesquiterpene lactone isolated from Antrodia cinnamomea, and its molecular mechanisms. Cancer Lett. 2016;373(2):174–184. 26679052 10.1016/j.canlet.2015.11.046

[26] Lin GL, Cohen DM, Desai RA, et al. Activation of beta 1 but not beta 3 integrin increases cell traction forces. FEBS Lett. 2013;587(6):763–769. 23395612 10.1016/j.febslet.2013.01.068 PMC3966909

[27] Schwander M, Leu M, Stumm M, et al. Beta1 integrins regulate myoblast fusion and sarcomere assembly. Dev Cell. 2003;4(5):673–685. 12737803 10.1016/s1534-5807(03)00118-7

[28] Lui C, Lee K, Nelson CM. Matrix compliance and RhoA direct the differentiation of mammary progenitor cells. Biomech Model Mechanobiol. 2012;11(8):1241–1249. 22161021 10.1007/s10237-011-0362-7 PMC3339284

[29] Maa MC, Chang MY, Hsieh MY, et al. Butyrate reduced lipopolysaccharide-mediated macrophage migration by suppression of Src enhancement and focal adhesion kinase activity. J Nutr Biochem. 2010;21(12):1186–1192. 20149623 10.1016/j.jnutbio.2009.10.004

[30] López-Colomé AM, Lee-Rivera I, Benavides-Hidalgo R, et al. Paxillin: a crossroad in pathological cell migration. J Hematol Oncol. 2017;10(1):50. 28214467 10.1186/s13045-017-0418-y PMC5316197

[31] Subauste MC, Pertz O, Adamson ED, et al. Vinculin modulation of paxillin-FAK interactions regulates ERK to control survival and motility. J Cell Biol. 2004;165(3):371–381. 15138291 10.1083/jcb.200308011 PMC2172187

[32] Wu J, He D, Yue B, et al. miR-101-1 expression pattern in Qinchuan cattle and its role in the regulation of cell differentiation. Gene. 2017;636:64–69. 28919162 10.1016/j.gene.2017.09.026

[33] Assoian RK, Schwartz MA. Coordinate signaling by integrins and receptor tyrosine kinases in the regulation of G1phase cell-cycle progression. Curr Opin Genet Dev. 2001;11(1):48–53. 11163150 10.1016/s0959-437x(00)00155-6

[34] Salasznyk RM, Klees RF, Williams WA, et al. Focal adhesion kinase signaling pathways regulate the osteogenic differentiation of human mesenchymal stem cells. Exp Cell Res. 2007;313(1):22–37. 17081517 10.1016/j.yexcr.2006.09.013 PMC1780174

[35] Sieg DJ, Hauck CR, Schlaepfer DD. Required role of focal adhesion kinase (FAK) for integrin-stimulated cell migration. J Cell Sci. 1999;112(Pt 16):2677–2691. 10413676 10.1242/jcs.112.16.2677

[36] Xu W, Coll JL, Adamson ED. Rescue of the mutant phenotype by reexpression of full-length vinculin in null F9 cells; effects on cell locomotion by domain deleted vinculin. J Cell Sci. 1998;111(Pt 11):1535–1544. 9580561 10.1242/jcs.111.11.1535

[37] Roberts WG, Ung E, Whalen P, et al. Antitumor activity and pharmacology of a selective focal adhesion kinase inhibitor, PF-562,271. Cancer Res. 2008;68(6):1935–1944. 18339875 10.1158/0008-5472.CAN-07-5155

